# Review of Intraocular Pharmacokinetics of Anti-Infectives Commonly Used in the Treatment of Infectious Endophthalmitis

**DOI:** 10.3390/pharmaceutics10020066

**Published:** 2018-05-29

**Authors:** Andrea Luaces-Rodríguez, Miguel González-Barcia, María José Blanco-Teijeiro, María Gil-Martínez, Francisco Gonzalez, Francisco Gómez-Ulla, María-Jesús Lamas, Francisco-Javier Otero-Espinar, Anxo Fernández-Ferreiro

**Affiliations:** 1 Department of Pharmacology, Pharmacy and Pharmaceutical Technology and Industrial Pharmacy Institute, Faculty of Pharmacy, University of Santiago de Compostela (USC), 15782 Santiago de Compostela, Spain; andrealuaces21@gmail.com (A.L.-R.); Miguel.gonzalez.barcia@segas.es (M.G.-B.); 2 Clinical Pharmacology Group, Health Research Institute of Santiago de Compostela (IDIS), 15706 Santiago de Compostela, Spain; Maria.Jesus.Lamas.Diaz@sergas.es; 3 Pharmacy Department, Clinical University Hospital Santiago de Compostela (SERGAS), 15706 Santiago de Compostela, Spain; 4 Ophthalmology Department, Clinical University Hospital Santiago de Compostela (SERGAS), 15706 Santiago de Compostela, Spain; mariajose.blanco@usc.es (M.J.B.-T.); mariagilmtez@hotmail.com (M.G.-M.); francisco.gonzalez@usc.es (F.G.); franciscogomez-ulla@institutogomez-ulla.es (F.G.-U.); 5 Department of Surgery and Medical-Surgical Specialties and CIMUS, University of Santiago de Compostela (USC), 15782 Santiago de Compostela, Spain

**Keywords:** anti-infectives, intravitreal, endophthalmitis, pharmacokinetics, infection

## Abstract

Although intravitreal administration of anti-infectives represents the standard treatment for infectious endophthalmitis, the knowledge about their pharmacokinetics is still limited. In this review, we aimed to summarise the factors influencing the pharmacokinetics of the anti-infective agents. We have conducted a comprehensive review of the preclinical pharmacokinetic parameters obtained in different studies of intravitreal injections of anti-infectives performed on animals, mainly rabbits. The two aspects with the biggest influence on pharmacokinetics are the distribution in the vitreous humour and the elimination through the posterior segment. The distribution can be affected by the molecular weight of the drug, the convection flow of the vitreous, the condition of the vitreous humour depending on the age of the patient, the possible interactions between the drug and the components of the vitreous, and the presence of vitrectomy. Meanwhile, the elimination includes the metabolism of the drug, the clearance via the anterior and posterior routes, and the possible inflammation of the eye resulting from the disease. Understanding the pharmacokinetics of the anti-infectives used in clinical practice is essential for a correct application. The information provided in this review could offer guidance for selecting the best therapeutic option according to the characteristics of the drugs.

## 1. Introduction: The Infectious Endophthalmitis and the Need for Anti-Infective Treatment

Endophthalmitis is severe inflammation involving both the anterior and posterior segments of the eye secondary, in the majority of cases, to an infectious agent, such as bacteria, fungi or, in isolated cases, to parasites. The majority of the time, the microorganisms are of exogenous origin and manage to penetrate the eye during surgical procedures, after the administration of intravitreal injections, or due to ocular trauma [1].

The severity and clinical course of the infectious endophthalmitis is related to the virulence and inoculation of infectious microorganisms, as well as to the delay in its diagnosis and the patient’s immune status. The infectious process starts with an initial incubation phase in which a critical load of microorganisms reaches the inside of the eye and begins to proliferate. It continues with production of fibrin and infiltration of neutrophils, followed by an acceleration phase which produces an immune response with numerous macrophages and lymphocytes infiltrating into the vitreous cavity and accumulating in the aqueous humour [2]. Finally, if not treated, it follows with a phase of tissue destruction.

The vitreous humour is a water-based gelatinous substance, rich in hyaluronic acid and collagen, which promotes the proliferation of microorganisms. The vitreous humour is not vascularized, and, as a consequence, the immune system is unable to control the presence of a microorganism inside the vitreous cavity [3]. For this reason, using anti-infectives capable of reaching the intraocular cavity and the surrounding ocular tissues is necessary in order to control the infection [3,4].

Treatment for the infectious endophthalmitis must be administered as soon as the disease is suspected, with an empirical broad-spectrum treatment upon suspicion of potential microorganisms acting as etiological agents. Once the microorganism or microorganisms causing the infection are known, anti-infective agents with a spectrum of action against such microorganisms must be selected. These agents must reach effective concentrations in the vitreous humour in order to eradicate the microorganisms as soon as possible. Otherwise, there is a very high risk of causing irreversible blindness [3] and, in many cases, the loss of the eye.

## 2. Possible Routes of Administration of Anti-Infective Agents in Infectious Endophthalmitis and Intravitreals Currently Available

**Figure 1:**
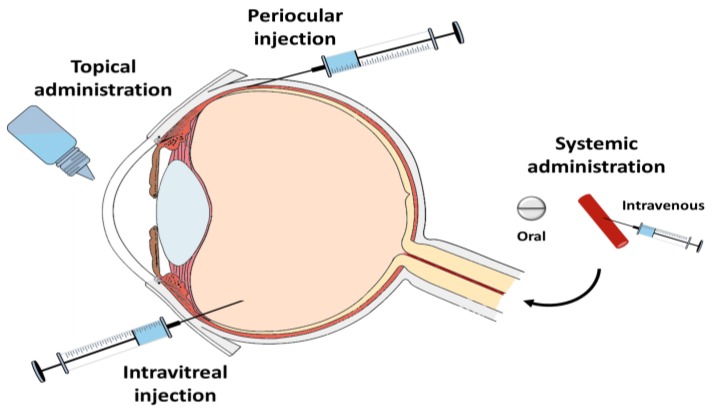
Scheme of the routes of administration of anti-infectives into the eye.

The possible routes of administration for anti-infectives, so they can reach the posterior segment of the eye, are the topical ophthalmic route, the systemic route, the intravitreal route and periocular administration [5] (Figure 1). Generally, topical and systemic ocular routes of administration of anti-infectives do not provide adequate concentrations of anti-infectives in the vitreous humour, due to various physiological factors [6].

Currently, the available information regarding the ocular penetration of drugs delivered via the systemic route is still limited [3]. In the majority of cases, effective concentrations of anti-infectives in the vitreous humour are not reached after oral or parenteral administration. It has been reported that only some fluoroquinolones (levofloxacin and moxifloxacin) [7,8,9,10] and linezolid [11,12,13] reach adequate levels in the vitreous humour via the systemic route in order to eliminate the microorganisms causing the infection [4].

The “Endophthalmitis Vitrectomy Study” conducted during the 1990s, found that a group of patients treated with systemic antibiotics showed no improvement regarding their disease [14]. Overall, the use of antibiotics via the parenteral route is not recommended for the treatment of endophthalmitis. This is because it promotes the appearance of systemic toxic effects, and antibiotics take too long to reach effective concentrations in the vitreous humour. The delay in reaching an effective concentration of anti-infectives in the vitreous humour could lead to permanent damage of the ocular tissues and cause blindness in the affected eye. However, in those cases of endogenous endophthalmitis, in which the source of infection is external to the eye cavity, patients would benefit from systemic antibiotic treatment [15].

The topical ocular administration of anti-infectives is the chosen route for the treatment of infectious diseases of the anterior segment of the eye. However, due to low ocular retention of the majority of ophthalmic formulations and poor drug penetration into the vitreous humour, anti-infectives via topical ocular route are not used for the treatment of infections of the posterior segment in clinical practice [16].

The intravitreal injection of anti-infectives is the delivery route that enables immediate high concentrations of drug inside the vitreous for a longer period of time. Table 1 shows the anti-infective agents which are most commonly used in clinical practice for intravitreal injections, as well as the used dosage.

The first administrations of intravitreal antibiotics were conducted in the 1940s, although it was not until the 1970s that this practice became widespread as a treatment for endophthalmitis. The safety and efficacy of various antibiotics delivered via intravitreal route were tested in rabbit eyes, which permitted to establish the appropriate dosage for human administration [25,26]. To this day, the intravitreal administration of anti-infectives represents the standard treatment for endophthalmitis, complemented in some cases with a vitrectomy in order to promote the eradication of microorganisms [27].

## 3. Factors Involved in the Intravitreal Pharmacokinetics of Anti-Infectives

Nowadays, the pharmacokinetics and pharmacodynamics of anti-infective agents after intravitreal administration remain relatively unexplored and poorly understood. The clearance mechanisms of anti-infectives of the vitreous humour limit the duration of their effect, thus requiring in some cases the repeated administration of injections in order to completely eliminate the infection [28]. The study of the pharmacokinetics of anti-infectives clearance in the vitreous humour will enable the optimization of the dosage guidelines for such treatments.

Once an anti-infective solution is injected into the vitreous humour, the initial concentration of the anti-infective in the vitreous cavity depends on the extent of its distribution and the initial dose. However, the concentration at any certain time after the injection depends on the distribution volume of the drug, the initial injected dose and the elimination rate. Therefore, the two factors predominantly affecting the pharmacokinetics of drugs injected into the vitreous are their distribution in the vitreous humour and their elimination [29,30].

### 3.1. Distribution of Anti-Infectives in the Vitreous

There are three parameters that will determine distribution velocity in the vitreous. The first is anti-infective diffusion in the matrix of the vitreous humour. The second is the effect of convection flow on anti-infective mobility. And the third is linked to the possible interactions that could be established between the drug and the various components of the vitreous humour.

#### 3.1.1. Influence of the Molecular Weight and the Charge of Anti-Infectives

Drugs will usually show a higher or lower diffusivity in the vitreous humour depending on their molecular weight [31]. Generally, drugs of a low molecular weight do not show any restrictions regarding diffusion; therefore, the diffusivity in an aqueous solution can be used as an accurate representation of the diffusivity of a molecule in the vitreous humour. This is possible as long as the drug does not interact with the components of the vitreous humour [32]. With regard to molecules of a higher molecular weight, their diffusivity seems to be limited by the structure of the vitreous humour [31].

Anti-infectives usually have a small molecular weight, lower than 500 Da, which is the estimated size of the vitreous humour meshwork [33], so the vitreous humour does not constitute a barrier for the diffusion of anti-infectives. In fact, the diffusivity of small molecules in the humour is relatively similar to their diffusion in water [16]. Consequently, it can be stated that anti-infectives spread rapidly in the vitreous humour, although their complete diffusion in it could take some hours [34].

Consequently, it can be deduced that the concentration reached in the vitreous humour shortly after the injection will be equal to the delivered dose divided by the volume of vitreous humour, which in humans is approximately 4 mL. As an example, a vancomycin injection of 1000 µg, the recommended dose for humans, will produce a concentration in the vitreous humour of 250 µg/mL. If we take into account that minimum inhibitory concentrations for the majority of sensitive microorganisms are between 1 and 5 µg/mL, we find that the doses delivered in clinical practice constitute a very high initial concentration of anti-infective [29].

#### 3.1.2. Influence of Vitreous Convection on the Distribution of Anti-Infectives

On the other hand, the convection flow that is induced in the vitreous humour also has an effect on the movement of the drugs in it. Convection is due to the fact that a fraction of the aqueous humour generated in the ciliary processes flows through the vitreous humour towards the retina [31,35,36].

The significance of the effect that convection in the vitreous humour has on the distribution of drugs depends on the diffusivity of said drugs in the vitreous. Convection does not affect the distribution in the vitreous of drugs with high diffusivity values (1 × 10^−5^ cm^2^/s) but it can become relevant for drugs with low diffusivity (1 × 10^−5^ cm^2^/s), particularly in cases with increased flow [36,37]. Increased flows in the vitreous can be observed in some conditions such as glaucoma or rhegmatogenous retinal detachment [35,36]. Anti-infectives do not have high molecular weights so they will show high diffusivity values, and, therefore, the relevance of convection flow will be low.

#### 3.1.3. Physiological and Pathological Conditions of the Vitreous

##### Influence of Patient Age and Vitreous Composition

One factor affecting both diffusion and convection in the vitreous humour is its liquefaction, which is the degeneration process of the vitreous humour associated with ageing. The vitreous humour consists of a vitreous humour in liquid form and a vitreous humour in gel form [38]. Ageing modifies the ratio between the two types, with the liquid increasing while the gel decreases [31]. This is due to a disruption in the meshwork of fibres that compose the vitreous. This liquefaction of the vitreous is responsible for an increase in drug diffusivity, particularly for those drugs that showed limited diffusion, such as high molecular weight drugs since there are less restrictions for the movement of molecules inside the meshwork of fibres. This increase in diffusion can lead to an increase in the elimination, although liquefaction in itself does not directly affect the elimination of drugs from the posterior segment of the eye. The higher the liquefaction of the vitreous humour, the more significant the resemblance between molecule diffusivity in the vitreous humour and molecule diffusivity in water [16]. On the other hand, liquefaction and loss of the homogeneity of the vitreous humour caused by ageing can also be linked to an increase in convection [34].

Such data mean that treating patients of different age groups with the same dosage scheme might be inappropriate, because it may lead to overdose or insufficient dosage situations. Even so, to this day, there is no evidence that the liquefaction of the vitreous humour may lead to a significant change in the pharmacokinetics of intravitreal drugs [39]. However, we must take into account that, since the majority of anti-infectives show high diffusivities, it is unlikely that liquefaction has a significant effect on their diffusion [16].

##### Interaction of Drugs with the Components of the Vitreous Humour

###### Proteins

Even though protein concentration within the vitreous humour is very low [40,41], the binding of drugs to these proteins is feasible. The binding of drugs to the vitreous proteins will lead to a slowdown in their diffusion.

The binding of various drugs to vitreous humour proteins has already been studied, and different binding values have been obtained depending on the type of antibiotic. The fraction of antibiotic not bound to protein was 95–99% for levofloxacin, 16–27% for meropenem, 36–82% for moxifloxacin, 58–89% for vancomycin and 99–100% for fosfomycin [42,43]. This could have an effect on local anti-infective activity within the vitreous humour because only the bound fraction is able to exert its action.

A study has proven that there is a small increase in the concentration of proteins in the vitreous humour in cases of diabetic vitreoretinopathy, mainly linked to inflammation and immunity [44]. However, there is no published data regarding whether this small increase in the amount of proteins could have an impact on the interaction with anti-infectives.

###### Vitreous Humour

The positively charged molecules deserve a special mention, since vitreous molecules are negatively charged and electrostatic interactions between the two of them may take place and this could lead to a decrease in drug diffusivity [16,45]. Table 1 shows the molecular weight and net charge at the pH of the vitreous humour, for the anti-infectives which are most commonly used via intravitreal route in clinical practice.

##### Consequences of the Vitrectomy

As previously it is necessary to perform a vitrectomy in some endophthalmitis cases, such as in patients with visual acuity of light perception or diagnostic vitrectomy, so as to enable the elimination of microorganisms. It has been found that the half-life of anti-infectives decreases when the vitreous humour has been removed. Table 2 shows the pharmacokinetic data obtained for vitrectomised eyes. This suggests that the vitreous humour meshwork is indeed important, because it retains the anti-infectives in it [30].

Theoretically, pars plana vitrectomy in endophthalmitis would allow for partial elimination of microorganisms, intraocular toxics and vitreous membranes, the extraction of diluted samples for microbiological study and a better distribution of intravitreal antibiotics.

Since the publication of the Endophthalmitis Vitrectomy Study (EVS) [14], randomised and of 2 × 2 factorial design, it was concluded that there was no benefit on the execution of vitrectomy unless visual acuity was impaired to light perception (LP). However, and since endophthalmitis is a dynamic process capable of making rapid progress, it seems reasonable to eliminate all of the damaging agents from the vitreous cavity and to do it before visual acuity is impaired to LP and the damage becomes irreversible. Performing a vitrectomy in endophthalmitis is particularly complex and the way in which it is performed implies diverse pharmacokinetic and anatomo-functional consequences for the eye. Visualization is compromised and the retina is very fragile, sometimes necrotic. In order to prevent iatrogenic retinal damage and the subsequent retinal detachment, the EVS protocol prohibited the induction of posterior vitreous detachment and limited the extension of the vitrectomy to 50% of the vitreous content, which meant that abundant purulent material remained in the posterior vitreous, in contact with the retinal surface.

Kuhn et al. [72] support the execution of a “complete” vitrectomy in anterior or posterior direction, with the use of temporal keratoprosthesis in cases of corneal opacity, clearing of the anterior chamber, posterior capsulotomy and premature induction of posterior vitreous detachment. In the cases where there is severe retinal damage, intraoperative retinal detachment or large areas of necrotic retina, silicone oil could be injected as a vitreous substitute [73].

In vitrectomised eyes there is a faster clearance of the anti-infectives that are eliminated via the posterior route. Besides, it has been claimed that the injection of anti-infectives into vitrectomised eyes may increase the risk of retinal toxicity. This could be due to the fact that the anti-infective gets deposited on the surface of the retina, causing it to come into contact with a high amount of anti-infective, instead of with an even distribution of the entire dose in the vitreous humour [74]. A reduction on in the dose could be considered (some authors would suggest 50%) if a complete vitrectomy has been performed, since the vitreous humour inhibits the rapid delivery of anti-infectives to the retina. This is extremely important in the case of amikacin, since it has been proven to produce retinal toxicity [75].

Moreover, it is important to consider that the use of sealants (gas/silicone oil), which is frequent in complex vitreoretinal surgery, alters the dosage and pharmacodynamics of intravitreal anti-infectives. Hegazy et al. [76] proved retinal toxicity at routinely used doses of intravitreal antibiotics in silicone oil-filled eyes. Hypothetically, retinal toxicity would occur due to the reduction of the preretinal space; the drug would be confined to the aqueous space surrounding the oil bubble, which would alter its distribution and prolong its elimination period. Thus, these authors recommended a substantial reduction of the dose (1/4–1/10 of the standard dose) of antibiotics in eyes containing gas or silicone oil. Another option for cases in which sealing is required would be internal irrigation with antibiotic, prior to the exchange [73].

#### 3.1.4. Drug-Drug Interactions in the Vitreous Humour

The anti-infective treatment is normally applied before the detection of the microorganism causing the endophthalmitis. Thus, an empiric treatment that covers Gram-positive and Gram-negative agents is used. A combination of drugs capable of covering both types of microorganisms is commonly administered and therefore, drug-drug interactions may occur within the vitreous humour. However, there is limited data regarding drug-drug interactions in the intraocular tissues due to the challenging conditions of these studies.

The standard treatment for bacterial endophthalmitis is the co-administration of 0.1 mg/0.1 mL vancomycin and 2 mg/0.1 mL ceftazidime [2,77]. However, a physical incompatibility between both drugs has been detected, as the concomitant administration of both causes ceftazidime and vancomycin precipitation [78]. Both the difference in pH of the two formulations and the presence of sodium bicarbonate have been reported to cause the precipitation [79]. Nevertheless, the vancomycin-cefatizidime combination remains effective and precipitates are removed from the vitreous humour approximately two months post-injection [79].

Ciprofloxacin is stable at a pH of around 4.5, but it starts precipitating if the pH increases to above 7. A study analysed the precipitation of ciprofloxacin in the vitreous humour due to concomitant vancomycin treatment. However, the conclusion was drawn that ciprofloxacin precipitation was not associated with the presence of vancomycin in the vitreous humour, and therefore a drug-drug interaction did not occur [80].

### 3.2. Elimination of Anti-Infectives from the Vitreous Humour

Another factor influencing intravitreal pharmacokinetics is drug clearance in the vitreous humour. Here, the metabolism experienced by drugs inside the vitreous humour must be taken into account, as well as the different existing elimination routes.

#### 3.2.1. Drug Metabolism in the Vitreous Humour

Drug metabolism in the vitreous humour has not been analysed in detail. Most of the work conducted in this field has focused on the identification of enzymes at the vitreous humour level, without relating them to their possible influence on pharmacokinetics [81]. On the other hand, it is worth mentioning the presence of enzymes such as esterases or peptidases in rabbit vitreous humour [82], which have been used for developing prodrugs, such as ganciclovir esters, which, once injected into the vitreous humour, biotransform into the drug ganciclovir [82].

Other ocular tissues, such as the retina, the ciliary body and the iris have also proven to contain enzymes involved in drug metabolism [83], but, in this case, the metabolism of drugs would be subsequent to their elimination from the vitreous cavity.

Cytochromes P450 are responsible for the biotransformation of most drugs in clinical use, making, whether or not this enzyme family is present in the ocular tissues, an interesting finding. A study demonstrated the presence of the CYP450 enzymes CYP2A6, CYP2C8, CYP2D6 and CYP2E1 in the retina/choroid by means of an mRNA expression analysis in human ocular tissues [84]. The limited mRNA expression levels found suggest that P450-mediated metabolism may contribute to the overall metabolism in the eye, but not significantly [84]. However, new studies are needed if we are to know the exact impact of CYP450 on the drug metabolism after intravitreal administration.

#### 3.2.2. Vitreous Clearance of Drugs

Regarding elimination routes, drugs can leave the vitreous cavity through the anterior or posterior route.

Although the anterior route is available for all drugs, hydrophilic molecules and compounds of a large size are mainly eliminated through this route [81]. This is based on drug diffusion through the vitreous humour towards the anterior chamber, where they access the aqueous humour. Drugs are subsequently eliminated through the Schlemm’s canal onto the general circulation. Drugs eliminated in this way have a half-life of less than 24 h [16,81].

The lens does not contribute to the elimination of drugs via the anterior route, but it does offer a barrier against their movement towards the anterior chamber. Because of this, the half-lives of drugs eliminated via the anterior route get reduced when the lens is removed [30,34].

The posterior route is based on the elimination of the drug from the posterior segment through the retina and towards the capillaries, and from there onto the systemic circulation. Drugs that are to be eliminated via this route must pass through a barrier which consists of the retinal capillaries and the pigment epithelium. This transport is primarily executed by means of passive diffusion for those compounds of a small size and with lipophilic properties, although an active transport also takes place [81]. The active transport enables the elimination of compounds with a high molecular weight through the retina [30].

Several pharmacokinetic studies in animal and human models have established that antibiotics such as vancomycin, aminoglycosides, macrolides and rifampicin tend to get eliminated through the anterior route [3,29,30]. However, antibiotics such as beta-lactams, clindamycin and fluoroquinolones tend to get eliminated through the posterior route [3,29,30]. It can also be the case that drugs are eliminated through both routes, and in this respect, antibiotics such as ceftriaxone, ceftazidime or ciprofloxacin seem to experience this double elimination process [30]. Table 2 presents the results of an extensive review which shows the preclinical pharmacokinetic parameters of some of the anti-infective drugs most commonly used in routine clinical practice, showing the elimination route as well as pathological or surgical conditions, which might affect the half-life of each one of them.

##### Transporters in the Blood Retinal Barrier (BRB)

The vitreous chamber is surrounded by the blood-ocular barriers, which consist of the anterior blood-aqueous barrier (BAB) and posterior blood-retinal barrier (BRB). The BAB is composed of the non-pigmented ciliary capillaries and the tight endothelia around the iris and ciliary muscle capillaries. The BRB is composed of the inner blood retinal barrier, which is in contact with the vitreous humour and consists of capillary endothelial cells connected by tight junctions, and the outer blood retinal barrier, also called retinal pigment endothelial (RPE) [16,31,85].

There is evidence of many influx and efflux transporters at the BRB level [31,85,86]. However, their expression is sometimes controversial, as many studies have been performed on animal models and its translation into humans is not always safe. Some of the detected transporters are MDR1 (P-glycoprotein), BCRP, MRP and OATP [87,88]. Some drugs can be substrates of the active transporters at the BRB level, but their contribution to drug pharmacokinetics is still unclear. However, the contribution of the active transporters to the drug movement through the BRB is expected to be quite low, as the concentration of the drug in the vitreous humour is very high and therefore the transporters should be saturated [87].

P-glycoprotein (P-gp) or multi-drug resistant 1 protein (MDR1) is one of the most studied efflux transporters at the blood brain barrier (BBB) level, and it is responsible for the poor penetration of many therapeutic drugs into the brain [89]. P-gp has been detected both in the apical and basal cell layers of human retinal pigment epithelium [90]. As it happens in the BBB, basolateral P-glycoprotein could have a protective function regarding the neural retina, helping to clear out unwanted substances [90]. However, compared to the BBB, P-glycoprotein seems to have a lesser effect on the permeability of the BRB [91,92,93].

Among the antibiotics recognised as substrates or modulators of P-gp, structurally unrelated compounds as fluoroquinolones, macrolides, ansamycines, tetracyclines and anthracyclines can be found [89]. Although there is some data on the effect of P-gp on these agents in different tissues, information about how P-gp can affect intraocular pharmacokinetics of antibiotics is still needed.

##### Influence on the Clearance of the Status of Ocular Inflammation

Lastly, it is necessary to point out that the status of ocular inflammation can affect, in varying degrees, drug clearance in the vitreous humour [45]. Drugs eliminated via the anterior route can see their elimination increased, although the mechanism causing this effect is unclear [30,81]. By contrast, drugs eliminated via the posterior route can see their elimination either increased or reduced, depending on whether they are eliminated by means of passive diffusion or active transport. It is believed that the status of ocular inflammation can lead to an increase in retinal permeability and vessel walls, which would explain the decrease in the half-life of drugs eliminated by means of passive diffusion [30,81].

### 3.3. Repetition Rate of Intravitreal Injections

Another aspect that will affect pharmacokinetics is the administration frequency of intravitreal anti-infectives. This frequency is generally based on average life and clinical response. The purpose of repeating the injections is to prolong the time during which the anti-infective is found in a concentration higher than the minimum inhibitory concentration (MIC). The administration of a single dose is usually preferred to repeat doses, and that is why the doses selected for administration are the highest ones possible within safe thresholds, so anti-infectives are above the MIC for as long as possible. However, it must be taken into account that a higher concentration, or an increase in the frequency of injection with a view to securing a concentration above the MIC for the whole period, could lead to an increase in the risk of adverse effects.

## 4. Conclusions

The understanding of the pharmacokinetics of the anti-infectives used in clinical practice may help to treat intraocular infections. Maintaining effective intravitreal concentrations during a prolonged period of time and avoiding side effects caused by high concentrations of anti-infective agents [79] should improve the outcome of this condition.

The use of individualized doses adjusted to the clinical characteristics of each patient is customary in the administration of drugs. For the estimation of these doses, factors such as renal function [94] or metabolism type of the patient are taken into account [95]. However, this practice does not apply to the field of ophthalmology, where standardized doses of anti-infectives are used, established in the majority of cases by means of empirical methods. This is because the number of pharmacokinetic studies available in this field is limited due to the fact that ophthalmological studies require invasive techniques.

With this review, the pharmacokinetic parameters of the anti-infective drugs which are most commonly used in routine clinical practice, as well as the factors that may have an effect on them, are analysed. The objective is that this information, provided just as a small contribution to the increasingly demanded personalized medicine, could serve to guide the clinician regarding the election of the best therapeutic option, according to the characteristics of each patient.

## Figures and Tables

**Table 1:** Anti-infective intravitreal injections for human use as outlined in the various ophthalmic formularies [17,18,19,20,21,22]. It shows the molecular weight of the active agents, as well as the electric charge they will have in contact with the vitreous. The molecular weight (MW) values of all of the compounds were extracted from the database The PubChem Project [23]. The charge of the anti-infectives at the pH of the vitreous humour (7.4) was calculated with the online platform Chemicalize [24], including the symbol (+) if the net charge is positive and (−) if the net charge is negative.

	MW	Net Charge
INTRAVITREAL ANTIBIOTICS		
Amikacin 0.4 mg/0.1 mL o 0.1 mg/0.1 mL	585.608 g/mol	+3.80
Ampicillin 5 mg/0.1 mL	349.405 g/mol	−0.60
Aztreonam 0.1 mg/0.1 mL	435.426 g/mol	−2.00
Cefazolin 2.25 mg/0.1 mL or 2.5 mg/0.1 mL	454.498 g/mol	−1.00
Cefotaxime 0.4 mg/0.1 mL	455.460 g/mol	−1.00
Ceftazidime 2 mg/0.1 mL	546.573 g/mol	−1.00
Ceftriaxone 2 mg/0.1 mL	554.571 g/mol	−2.00
Ciprofloxacin 0.1 mg/0.1 mL	331.347 g/mol	−0.02
Clindamycin 0.5 mg/0.1 mL and 1 mg/0.1 mL	424.981 g/mol	+0.59
Gentamicin 200 μg/0.1 mL	477.603 g/mol	+4.52
Levofloxacin 0.625 mg/0.1 mL	361.373 g/mol	−0.92
Lincomycin 1 mg/0.1mL	406.538 g/mol	+0.79
Moxifloxacin 160 μg /0.1 mL	401.438 g/mol	+0.01
Penicillin G 300 units/0.1 mL	334.390 g/mol	−1.00
Piperacillin/Tazobactam 1.5 mg/0.1 mL	517.557 g/mol/300.289 g/mol	−1.00
Tobramycin 100 μg /0.1 mL or 200 μg /0.1 mL or 300 μg /0.1 mL or 400 μg /0.1 mL	467.520 g/mol	+4.42
Vancomycin 1 mg/0.1 mL or 2 mg/0.1 mL	1449.265 g/mol	+0.89
INTRAVITREAL ANTIFUNGALS		
Colloidal Amphotericin B 5 μg/0.1 mL	924.091 g/mol	−0.02
Voriconazole 0.05 mg/0.1 mL	349.317 g/mol	−0.00
INTRAVITREAL ANTIVIRALS		
Ganciclovir 20 mg/mL	255.234 g/mol	−0.00
Acyclovir 80 μg/0.1 mL or 200 μg/0.1 mL	225.208 g/mol	−0.00
Foscarnet 1220 μg/0.1 mL	126.004 g/mol	−2.06
Cidovofir 0.2 mg/mL and 8.1 mg/mL	279.189 g/mol	−1.38

**Table 2:** Preclinical pharmacokinetic parameters of the intravitreal injection of different anti-infectives in animal models.

Anti-Infective	Study Model	Delivered Dose in 0.1 mL	Elimination Route	t_1/2_ Normal Condition	t_1/2_ Inflammation	t_1/2_ Aphakia	t_1/2_ Aphakia + Vitrectomy	Ref.
ANTIBIOTICS								
Amikacin	Rabbit	400 µg/0.1 mL	Anterior	25.5 h	15.5 h	14.3 h	7.9 h	[46]
Aztreonam	Rabbit	100 µg	Posterior	7.5 h				[47]
Carbenicillin	Rabbit	1000 µg	Posterior	5 h	6 h			[48]
	Rhesus monkey	1000 µg	Posterior	10 h				[49]
Cefazolin	Rhesus monkey	1000 µg	Posterior	7 h				[49]
	Rabbit	2250 µg	Posterior	6.5 h	10.4 h	8.3 h	6.0 h	[50]
Cefepime	Rabbit	1000 µg	Anterior	14.3 h	15.1 h			[51]
Ceftazidime	Rabbit	1000 µg	Anterior	20 h	21.5 h			[51]
	Rabbit	2250 µg	Both	13.8 h	10.1 h	11.8 h	4.7 h	[52]
	Rabbit	1000 µg		8.1 h	11.7 h			[53]
Ceftriaxone	Rabbit	1000 µg	Both	9.1 h	13.1 h			[51]
Ciprofloxacin	Rabbit	250 µg		4.5 h				[54]
	Rabbit	100 µg	Posterior	2.2 h			1 h	[55]
	Rabbit	200 µg		6.02 h	15.06 h			[56]
Clarithromycin	Rabbit	1000 µg		2 h				[57]
Clindamycin	Rabbit	800 µg		3 h				[58]
Daptomycin	Rabbit	200 µg *	Both	25.67 h	34.6 h			[59]
Gentamicin	Rhesus monkey	100 µg	Anterior	33 h				[49]
Linezolid	Rabbit	1, 10, 30 mg			2 h			[60]
Moxifloxacin	Rabbit	200 µg	Posterior	1.72 h				[61]
Ofloxacin	Rabbit	200 µg		5.65 h	9.72 h			[62]
Vancomycin	Rabbit	1000 µg		25.1 h		8.9 h	9.0 h	[63]
	Rabbit	1000 µg	Both	62.34 h	14.53 h			[64]
	Rabbit	1000 µg		56 h	48 h			[65]
	Rabbit	500 µg			12.3 h			[66]
ANTIFUNGALS								
Amphotericin B	Rabbit	10 µg	Posterior	9.1 days	8.6 days	4.7 days	1.4 days	[67]
	Rabbit	9.1–13.4 µg		6.9–15.1 days			1.8 days	[68]
Caspofungin	Rabbit	50 µg	Posterior	6.28 h				[69]
Fluconazole	Rabbit	100 µg	Posterior	23 min 3.18 h				[70]
Voriconazole	Rabbit	35 µg	Posterior	2.5 h				[71]

* 200 µg in 0.05 mL.

## References

[1] Vaziri K., Schwartz S.G., Kishor K., Flynn H.W. (2015). Endophthalmitis: State of the art. Clin. Ophthalmol..

[2] Barry P., Cordovés L., Gardner S. (2013). ESCRS Guidelines for Prevention and Treatment of Endophthalmitis Following Cataract Surgery: Data, Dilemmas and Conclusions.

[3] López-Cabezas C., Muner D.S., Massa M.R., Mensa Pueyo J.M. (2010). Antibiotics in endophthalmitis: Microbiological and pharmacokinetic considerations. Curr. Clin. Pharmacol..

[4] Radhika M., Mithal K., Bawdekar A., Dave V., Jindal A., Relhan N., Albini T., Pathengay A., Flynn H.W. (2014). Pharmacokinetics of intravitreal antibiotics in endophthalmitis. J. Ophthalmic Inflamm. Infect..

[5] Barcia M.G. (2011). Formulación Magistral en Oftalmología. Aspectos Prácticos de la Farmacotécnia en un Servicio de Farmacia.

[6] Fernández-Ferreiro A., Barcia M.G. (2014). Patología ocular. Guía Rápida de Farmacia Hospitalaria.

[7] García-Sáenz M.C., Arias-Puente A., Fresnadillo-Martinez M.J., Carrasco-Font C. (2001). Human aqueous humor levels of oral ciprofloxacin, levofloxacin, and moxifloxacin. J. Cataract Refract. Surg..

[8] Fiscella R.G., Nguyen T.K., Cwik M.J., Phillpotts B.A., Friedlander S.M., Alter D.C., Shapiro M.J., Blair N.P., Gieser J.P. (1999). Aqueous and vitreous penetration of levofloxacin after oral administration. Ophthalmology.

[9] Hariprasad S.M., Shah G.K., Mieler W.F., Feiner L., Blinder K.J., Holekamp N.M., Gao H., Prince R.A. (2006). Vitreous and aqueous penetration of orally administered moxifloxacin in humans. Arch. Ophthalmol..

[10] Kampougeris G., Antoniadou A., Kavouklis E., Chryssouli Z., Giamarellou H. (2005). Penetration of moxifloxacin into the human aqueous humour after oral administration. Br. J. Ophthalmol..

[11] Ciulla T.A., Comer G.M., Peloquin C., Wheeler J. (2005). Human vitreous distribution of linezolid after a single oral dose. Retina.

[12] Fiscella R.G., Lai W.W., Buerk B., Khan M., Rodvold K.A., Pulido J.S., Labib S., Shapiro M.J., Blair N.P. (2004). Aqueous and vitreous penetration of linezolid (Zyvox) after oral administration. Ophthalmology.

[13] Horcajada J.P., Atienza R., Sarasa M., Soy D., Adán A., Mensa J. (2009). Pharmacokinetics of linezolid in human non-inflamed vitreous after systemic administration. J. Antimicrob. Chemother..

[14] Endophthalmitis Vitrectomy Study Group (1995). Results of the Endophthalmitis Vitrectomy Study. A randomized trial of immediate vitrectomy and of intravenous antibiotics for the treatment of postoperative bacterial endophthalmitis. Arch. Ophthalmol..

[15] Meredith T.A. (2006). Endophthalmitis. Intraocular Drug Delivery.

[16] Del Amo E.M., Rimpelä A.-K., Heikkinen E., Kari O.K., Ramsay E., Lajunen T., Schmitt M., Pelkonen L., Bhattacharya M., Richardson D. (2017). Pharmacokinetic aspects of retinal drug delivery. Prog. Retin. Eye Res..

[17] Moorfields Eye Hospital (NHS) (2017). Ophthalmic Formulary.

[18] McElhiney L.F. (2013). Compounding Guide for Ophthalmic Preparations.

[19] Herreros J.M.A. (2003). Preparación de Medicamentos y Formulación Magistral Para Oftalmología.

[20] Trissel L.A. (2012). A Trissel’s Stability of Compounded Formulations.

[21] Pérez-Santonja J.J., Hervás-Hernandis J.M. (2006). Queratitis Infecciosas: Fundamentos, Técnicas Diagnósticas y Tratamiento.

[22] Garg A., Sheppard J.D., Donnenfeld E.D., Friedlaender M.H. (2007). Clinical Applications of Antibiotics and Anti-Inflammatory Drugs in Ophthalmology.

[23] The PubChem Project. https://pubchem.ncbi.nlm.nih.gov/.

[24] Chemicalize. https://chemicalize.com/#/.

[25] Peyman G.A. (1977). Antibiotic administration in the treatment of bacterial endophthalmitis. II. Intravitreal injections. Surv. Ophthalmol..

[26] Baum J., Peyman G.A., Barza M. (1982). Intravitreal administration of antibiotic in the treatment of bacterial endophthalmitis. III. Consensus. Surv. Ophthalmol..

[27] Thomas B.J., Mehta N., Yonekawa Y., Sridhar J., Kuriyan A.E., Relhan N., Liang M.C., Woodward M.A., Witkin A.J., Shah C. (2017). Pars plana vitrectomy for late vitreoretinal sequelae of infectious endophthalmitis. Retina.

[28] Diakonis V.F., Tsourdou A., Tzatzarakis M.N., Tsika C., Charisis S., Naoumidi I., Plainis S., Tsilimbaris M.K. (2013). Evaluation of vitreous clearance and potential retinal toxicity of intravitreal lornoxicam (xefo). J. Ocul. Pharmacol. Ther..

[29] Meredith T.A. (2006). Intravitreal Antimicrobials. Intraocular Drug Delivery.

[30] Meredith T.A. (1993). Antimicrobial pharmacokinetics in endophthalmitis treatment: Studies of ceftazidime. Trans. Am. Ophthalmol. Soc..

[31] Wilson C.G., Tan L.E., Mains J. (2011). Principles of Retinal Drug Delivery from Within the Vitreous. Drug Product Development for the Back of the Eye.

[32] Friedrich S., Saville B., Cheng Y.L. (1997). Drug distribution in the vitreous humor of the human eye: The effects of aphakia and changes in retinal permeability and vitreous diffusivity. J. Ocul. Pharmacol. Ther..

[33] Xu Q., Boylan N.J., Suk J.S., Wang Y.-Y., Nance E.A., Yang J.-C., McDonnell P.J., Cone R.A., Duh E.J., Hanes J. (2013). Nanoparticle diffusion in, and microrheology of, the bovine vitreous ex vivo. J. Control. Release.

[34] Maurice D.M. (2012). Mishima Ocular Pharmacokinetics. Pharmacology of the Eye.

[35] Krishnamoorthy M.K., Park J., Augsburger J.J., Banerjee R.K. (2008). Effect of retinal permeability, diffusivity, and aqueous humor hydrodynamics on pharmacokinetics of drugs in the eye. J. Ocul. Pharmacol. Ther..

[36] Park J., Bungay P.M., Lutz R.J., Augsburger J.J., Millard R.W., Sinha Roy A., Banerjee R.K. (2005). Evaluation of coupled convective-diffusive transport of drugs administered by intravitreal injection and controlled release implant. J. Control. Release.

[37] Stay M.S., Xu J., Randolph T.W., Barocas V.H. (2003). Computer simulation of convective and diffusive transport of controlled-release drugs in the vitreous humor. Pharm. Res..

[38] Chirila T.V., Hong Y. (2016). Chapter C2 The Vitreous Humor.

[39] Del Amo E.M., Urtti A. (2015). Rabbit as an animal model for intravitreal pharmacokinetics: Clinical predictability and quality of the published data. Exp. Eye Res..

[40] Angi M., Kalirai H., Coupland S.E., Damato B.E., Semeraro F., Romano M.R. (2012). Proteomic Analyses of the Vitreous Humour. Mediat. Inflamm..

[41] Murthy K.R., Goel R., Subbannayya Y., Jacob H.K., Murthy P.R., Manda S.S., Patil A.H., Sharma R., Sahasrabuddhe N.A., Parashar A. (2014). Proteomic analysis of human vitreous humor. Clin. Proteom..

[42] Schauersberger J., Jager W. (2002). In-vitro Investigation of the Protein Binding of Different Antibiotics in the Human Vitreous. Investig. Ophthalmol. Vis. Sci..

[43] Petternel V., Krepler K., Schauersberger J., Wedrich A. (2004). Fosfomycin in human vitreous: -In-vitro investigation of the protein binding of fosfomycin in human vitreous –Fosfomycin levels in the vitreous cavity after intravenous administration. Investig. Ophthalmol. Vis. Sci..

[44] Loukovaara S., Nurkkala H., Tamene F., Gucciardo E., Liu X., Repo P., Lehti K., Varjosalo M. (2015). Quantitative Proteomics Analysis of Vitreous Humor from Diabetic Retinopathy Patients. J. Proteome Res..

[45] Fernández-Ferreiro A., Luaces-Rodríguez A., Aguiar P., Pardo-Montero J., González-Barcia M., García-Varela L., Herranz M., Silva-Rodríguez J., Gil-Martínez M., Bermúdez M.A. (2017). Preclinical PET Study of Intravitreal Injections. Investig. Ophthalmol. Vis. Sci..

[46] Mandell B.A., Meredith T.A., Aguilar E., el-Massry A., Sawant A., Gardner S. (1993). Effects of inflammation and surgery on amikacin levels in the vitreous cavity. Am. J. Ophthalmol..

[47] Barza M., McCue M. (1983). Pharmacokinetics of aztreonam in rabbit eyes. Antimicrob. Agents Chemother..

[48] Barza M., Kane A., Baum J. (1982). The effects of infection and probenecid on the transport of carbenicillin from the rabbit vitreous humor. Investig. Ophthalmol. Vis. Sci..

[49] Barza M., Kane A., Baum J. (1983). Pharmacokinetics of intravitreal carbenicillin, cefazolin, and gentamicin in rhesus monkeys. Investig. Ophthalmol. Vis. Sci..

[50] Ficker L., Meredith T.A., Gardner S., Wilson L.A. (1990). Cefazolin levels after intravitreal injection. Effects of inflammation and surgery. Investig. Ophthalmol. Vis. Sci..

[51] Barza M., Lynch E., Baum J.L. (1993). Pharmacokinetics of newer cephalosporins after subconjunctival and intravitreal injection in rabbits. Arch. Ophthalmol..

[52] Shaarawy A., Meredith T.A., Kincaid M., Dick J., Aguilar E., Ritchie D.J., Reichley R.M. (1995). Intraocular injection of ceftazidime. Effects of inflammation and surgery. Retina.

[53] Waga J., Nilsson-Ehle I., Ljungberg B., Skarin A., Ståhle L., Ehinger B. (1999). Microdialysis for pharmacokinetic studies of ceftazidime in rabbit vitreous. J. Ocul. Pharmacol. Ther..

[54] Rootman D.S., Savage P., Hasany S.M., Chisholm L., Basu P.K. (1992). Toxicity and pharmacokinetics of intravitreally injected ciprofloxacin in rabbit eyes. Can. J. Ophthalmol..

[55] Pearson P.A., Hainsworth D.P., Ashton P. (1993). Clearance and distribution of ciprofloxacin after intravitreal injection. Retina.

[56] Oztürk F., Kortunay S., Kurt E., Ilker S.S., Inan U.U., Basci N.E., Bozkurt A., Kayaalp O. (1999). Effects of trauma and infection on ciprofloxacin levels in the vitreous cavity. Retina.

[57] Unal M., Peyman G.A., Liang C., Hegazy H., Molinari L.C., Chen J., Brun S., Tarcha P.J. (1999). Ocular toxicity of intravitreal clarithromycin. Retina.

[58] Fiscella R., Peyman G.A., Fishman P.H. (1987). Duration of therapeutic levels of intravitreally injected liposome-encapsulated clindamycin in the rabbit. Can. J. Ophthalmol..

[59] Ozcimen M., Sakarya Y., Ozcimen S., Sakarya R., Goktas S., Iyisoy S., Alpfidan I., Erdogan E. (2015). Clearance of intravitreal daptomycin in uveitis-induced rabbit model. Curr. Eye Res..

[60] Saleh M., Lefèvre S., Acar N., Bourcier T., Marcellin L., Prévost G., Subilia A., Gaucher D., Jehl F. (2012). Efficacy of intravitreal administrations of linezolid in an experimental model of S. aureus-related endophthalmitis. Investig. Ophthalmol. Vis. Sci..

[61] Iyer M.N., He F., Wensel T.G., Mieler W.F., Benz M.S., Holz E.R. (2006). Clearance of intravitreal moxifloxacin. Investig. Ophthalmol. Vis. Sci..

[62] Oztürk F., Kortunay S., Kurt E., Ubeyt Inan U., Sami Ilker S., Basci N.E., Bozkurt A., Oguz Kayaalp S. (1999). Ofloxacin levels after intravitreal injection. Effects of trauma and inflammation. Ophthalmic Res..

[63] Aguilar H.E., Meredith T.A., el-Massry A., Shaarawy A., Kincaid M., Dick J., Ritchie D.J., Reichley R.M., Neisman M.K. (1995). Vancomycin levels after intravitreal injection. Effects of inflammation and surgery. Retina.

[64] Coco R.M., López M.I., Pastor J.C., Nozal M.J. (1998). Pharmacokinetics of intravitreal vancomycin in normal and infected rabbit eyes. J. Ocul. Pharmacol. Ther..

[65] Park S.S., Vallar R.V., Hong C.H., von Gunten S., Ruoff K., D’Amico D.J. (1999). Intravitreal dexamethasone effect on intravitreal vancomycin elimination in endophthalmitis. Arch. Ophthalmol..

[66] Coco R.M., Lopez M.I., Pastor J.C. (2000). Pharmacokinetics of 0.5 mg of a single and a multiple dose of intravitreal vancomycin in infected rabbit eyes. J. Ocul. Pharmacol. Ther..

[67] Doft B.H., Weiskopf J., Nilsson-Ehle I., Wingard L.B. (1985). Amphotericin clearance in vitrectomized versus nonvitrectomized eyes. Ophthalmology.

[68] Wingard L.B., Zuravleff J.J., Doft B.H., Berk L., Rinkoff J. (1989). Intraocular distribution of intravitreally administered amphotericin B in normal and vitrectomized eyes. Investig. Ophthalmol. Vis. Sci..

[69] Shen Y.-C., Liang C.-Y., Wang C.-Y., Lin K.-H., Hsu M.-Y., Yuen H.-L., Wei L.-C. (2014). Pharmacokinetics and safety of intravitreal caspofungin. Antimicrob. Agents Chemother..

[70] Gupta S.K., Velpandian T., Dhingra N., Jaiswal J. (2000). Intravitreal pharmacokinetics of plain and liposome-entrapped fluconazole in rabbit eyes. J. Ocul. Pharmacol. Ther..

[71] Shen Y.-C., Wang M.-Y., Wang C.-Y., Tsai T.-C., Tsai H.-Y., Lee Y.-F., Wei L.-C. (2007). Clearance of intravitreal voriconazole. Investig. Ophthalmol. Vis. Sci..

[72] Kuhn F., Gini G. (2005). Ten years after... are findings of the Endophthalmitis Vitrectomy Study still relevant today?. Graefes Arch. Clin. Exp. Ophthalmol..

[73] Aras C., Ozdamar A., Karacorlu M., Ozkan S. (2001). Silicone oil in the surgical treatment of endophthalmitis associated with retinal detachment. Int. Ophthalmol..

[74] Peyman G.A., Vastine D.W., Raichand M. (1978). Experimental Aspects and Their Clinical Application. Ophthalmology.

[75] Campochiaro P.A., Lim J.I. (1994). Aminoglycoside toxicity in the treatment of endophthalmitis. The Aminoglycoside Toxicity Study Group. Arch. Ophthalmol..

[76] Hegazy H.M., Kivilcim M., Peyman G.A., Unal M.H., Liang C., Molinari L.C., Kazi A.A. (1999). Evaluation of toxicity of intravitreal ceftazidime, vancomycin, and ganciclovir in a silicone oil-filled eye. Retina.

[77] Wakely L., Sheard R. (2014). Recent Advances in Endophthalmitis Management.

[78] Fiscella R.G. (1993). Physical incompatibility of vancomycin and ceftazidime for intra-vitreal injection. Arch. Ophthalmol..

[79] Lifshitz T., Lapid-Gortzak R., Finkelman Y., Klemperer I. (2000). Vancomycin and ceftazidime incompatibility upon intravitreal injection. Br. J. Ophthalmol..

[80] Hui M., Kwok A.K.H., Pang C.P., Cheung S.W., Chan R.C.Y., Lam D.S.C., Cheng A.F.B. (2004). An in vitro study on the compatibility and precipitation of a combination of ciprofloxacin and vancomycin in human vitreous. Br. J. Ophthalmol..

[81] Ashton P. (2006). Retinal Drug Delivery. Intraocular Drug Delivery.

[82] Dias C.S., Anand B.S., Mitra A.K. (2002). Effect of mono- and di-acylation on the ocular disposition of ganciclovir: Physicochemical properties, ocular bioreversion, and antiviral activity of short chain ester prodrugs. J. Pharm. Sci..

[83] Duvvuri S., Majumdar S., Mitra A.K. (2004). Role of metabolism in ocular drug delivery. Curr. Drug Metab..

[84] Zhang T., Xiang C.D., Gale D., Carreiro S., Wu E.Y., Zhang E.Y. (2008). Drug Transporter and Cytochrome P450 mRNA Expression in Human Ocular Barriers: Implications for Ocular Drug Disposition. Drug Metab. Dispos..

[85] Cunha-Vaz J.G. (2004). The blood-retinal barriers system. Basic concepts and clinical evaluation. Exp. Eye Res..

[86] Mannermaa E., Vellonen K.-S., Urtti A. (2006). Drug transport in corneal epithelium and blood-retina barrier: Emerging role of transporters in ocular pharmacokinetics. Adv. Drug Deliv. Rev..

[87] Vellonen K.-S., Hellinen L., Mannermaa E., Ruponen M., Urtti A., Kidron H. (2017). Expression, activity and pharmacokinetic impact of ocular transporters. Adv. Drug Deliv. Rev..

[88] Chen P., Chen H., Zang X., Chen M., Jiang H., Han S., Wu X. (2013). Expression of efflux transporters in human ocular tissues. Drug Metab. Dispos..

[89] Lin J.H., Yamazaki M. (2003). Role of P-glycoprotein in pharmacokinetics: Clinical implications. Clin. Pharmacokinet..

[90] Kennedy B.G., Mangini N.J. (2002). P-glycoprotein expression in human retinal pigment epithelium. Mol. Vis..

[91] Chapy H., Saubaméa B., Tournier N., Bourasset F., Behar-Cohen F., Declèves X., Scherrmann J.-M., Cisternino S. (2016). Blood-brain and retinal barriers show dissimilar ABC transporter impacts and concealed effect of P-glycoprotein on a novel verapamil influx carrier. Br. J. Pharmacol..

[92] Fujii S., Setoguchi C., Kawazu K., Hosoya K. (2014). Impact of P-glycoprotein on blood-retinal barrier permeability: Comparison of blood-aqueous humor and blood-brain barrier using mdr1a knockout rats. Investig. Ophthalmol. Vis. Sci..

[93] Bauer M., Karch R., Tournier N., Cisternino S., Wadsak W., Hacker M., Marhofer P., Zeitlinger M., Langer O. (2017). Assessment of P-Glycoprotein Transport Activity at the Human Blood-Retina Barrier with (R)-11C-Verapamil PET. J. Nucl. Med..

[94] Thamlikitkul V., Dubrovskaya Y., Manchandani P., Ngamprasertchai T., Boonyasiri A., Babic J.T., Tam V.H. (2017). Dosing and Pharmacokinetics of Polymyxin B in Patients with Renal Insufficiency. Antimicrob. Agents Chemother..

[95] Barbarino J.M., Owusu Obeng A., Klein T.E., Altman R.B. (2017). PharmGKB summary: Voriconazole pathway, pharmacokinetics. Pharmacogenet. Genom..

